# Engaging Sustainable Reforestation and Forest Protection in the Wallacea Line, Indonesia

**DOI:** 10.12688/f1000research.159731.2

**Published:** 2025-06-06

**Authors:** Yohanis Ngongo, Gerson N. Njurumana, Markus Kudeng Sallata, Merryana Kiding Allo, Nurhaedah Muin, Wahyudi Isnan, Nardy Noerman Najib, Achmad Rizal Hak Bisjoe, Indra A. S. L. P. Putri, Hariany Siappa, Ronald T. P. Hutapea, Yelin Adalina, Suhartati Suhartati, Yohanes Leki Seran, Agustinus Panusunan Tampubolon

**Affiliations:** 1 Research Centre for Ecology and Ethnobiology, National Research and Innovation Agency, Cibinong, Bogor, West Java, 16911, Indonesia; 2 Research Centre for Macro Economic and Financial, National Research and Innovation Agency, Jakarta, Jakarta, Indonesia; 3 Research Centre for Biomass and Bioproducts, National Research and Innovation Agency, Cibinong, Bogor, West Java, Indonesia; 4 Research Centre for Applied Botany, National Research and Innovation Agency, Cibinong, Bogor, West Java, Indonesia; 5 Research Center for Behavioral and Circular Economy, National Research and Innovation Agency, Jakarta, Jakarta, Indonesia

**Keywords:** Wallace Line, forest protection, reforestation, community forestry, forest management unit, ecosystem services

## Abstract

The Wallacea region of Indonesia has high biodiversity and highly unique and endangered species. Its terrestrial ecosystems have unique flora and fauna found nowhere else. Nevertheless, the strategy for protecting and conserving the Wallacea ecosystem is like that in other parts of Indonesia, since it refers to the national forest and environmental regulations. The uniqueness of the Wallacea ecosystem does not reflect the extraordinary efforts of protecting and conserving the region’s pristine ecosystem. The continuing decline of the forestland and expansions of agricultural lands have indicated the need for a more fundamental and integrative approach to conserving and protecting the Wallacea ecosystem, particularly forestlands.

We use the actor-centered power (ACP) approach or ideas and use the Wallacea Line to highlight how this idea is contested and confronted with the dynamics of complex societies and ecosystems. The ACP approach is the most widely used one in the implementation of the community forest (CF) program in Indonesia. The CF program is one of Indonesia’s community-based forest management schemes that empowers local communities to manage state forests sustainably. We chose two national parks established in the Wallacea region, Mutis on Timor Island and Matalawa on Sumba Island, to elaborate further on the development, conservation, and changes that occurred within that landscape.

The ACP approach, in line with the spirit of the decentralization era, has mixed consequences for forest management and the biodiversity of the Wallacea region. Regarding the specific characteristics of the Wallacea region and lessons learned from the ACP approach implementation in the CF program, we then propose a sustainable model of reforestation and forest protection that applies the principle of “unity in diversity,” where all actors involved have space for the growth of creativity and positive contributions to sustainable forest protection.

## Introduction

Community forestry (CF) refers to the forest management undertaken by local communities either on their own or on leased private lands, communal lands, or state lands ( Wiersum, 2004). It is a set of institutional arrangements in which communities are involved, either wholly or in part, in decision-making processes for healthy forests and better social well-being ( Danks & Fortmann, 2004). In Indonesia, a community forest is a state forest in which communities undertake active roles in forest protection and sustainable forest management. The institutional arrangement of the CF is under central government regulation ( PP No. 6/2007, 2007), and there are more details under the regulation of the Ministry of Environment and Forestry Republic of Indonesia regarding community forestry ( Permenhut 88/2014, 2014).

Soon after the New Order government collapsed following major financial crises, the State control of forest management weakened, leading to fast deforestation ( Barr, 2001). Community forests in Indonesia were introduced following the spirit of the decentralization era, a decade after the Suharto regime of the New Order government collapsed in 1997. The actor-centered power (ACP) framework is the most widely used approach to returning power to the local people in managing forest resources. However, Krott et al. (2014, p.34) noted, “…, but some of the local and even extra-local elites acquired dominant influence and proceeded to misuse the community forest for their own.” The ACP framework is also used in other sectors, such as agriculture and rural development, or in natural resource-based development to mobilize and empower local actors to participate and work together in a chain or network to achieve a specific outcome ( Fisher et al., 2017; Juniyanti et al., 2021), enhance collaboration and social learning ( Chervier et al., 2020), and scale up to enhance farmers’ adaptive capabilities ( Pircher et al., 2022).

The collapse of the New Order regime has created an unclear situation for national government actors in forest management, while local actors have little experience in forest management or do not consider national forest management as much ( Colchester et al., 2014; McCarthy, 2000) and focus more on the euphoria surrounding the return of power and the extraction of natural resources. The devolution era offers good governance that minimizes corruption and is inclusive in the decision-making process ( Dasgupta & Roy, 2011). However, local governments in the devolution era have been more concerned about increasing revenue and less about forest protection and sustainability ( Hidayat, 2016). Forest destruction related to the decentralization era lies in the power of bureaucrat elites in issuing approval for land concessions and investment projects, or what the Critical Ecosystem Partnership Fund (2014, p.113) called “rent-seeking behavior among local officials and politicians.” The capitalist mode of production has led to forest conversion to large-scale commercial crop plantations, especially for oil palm ( Colchester et al., 2014). In the decentralization era, local communities and civil society actors have competed to claim the land rights for extracting forest resources ( Libert-Amico & Larson, 2020).

The good governance proposed in the devolution era is a strategy for better natural resource management; however, there has been limited support from local governments (provincial and district levels) in the implementation of forest-related programs, particularly the social forestry program ( Rahayu et al., 2024). There has been a power imbalance, where the execution of the program is biased toward government institutions ( Rahayu et al., 2024), and this can be overcome by the presence of local facilitators ( Arai et al., 2021). Forest governance is a process carried out by actors, powers, and rules, where the government, as the dominant actor, enforces regulatory mechanisms and guides the interaction and participation of actors ( Ramadhan et al., 2023). These actors play a role in using their power to result in policy changes in forest management. Policy improvements in several key aspects of forest governance can affect land cover change that allows deforestation control ( Nurrochmat et al., 2023). In addition, the delegation of power from dominant to lower-level actors is key in controlling deforestation ( Rochmayanto et al., 2023).

The ACP framework is widely implemented in Indonesia’s forest and natural resource management, including in Wallacea’s biogeography region. The Wallacea region covers Sulawesi, Maluku, and the Nusa Tenggara Islands, which have rich biodiversity and endemic species. Nevertheless, the region is threatened by biodiversity loss due to severe deforestation, which reached 10,231 km ^2^ within two decades and is estimated to reach 49,570 km ^2^ by 2053 ( Voigt et al., 2021). Even though the deforestation rate is decreasing, it will still occur until 2057 ( Nurrochmat et al., 2023). Deforestation in these areas can threaten endemic species’ natural habitats and cause significant biodiversity loss. The alarming rate of deforestation is related to forest governance in Indonesia, which has not run optimally ( Basu & Basu, 2023; Yuza et al., 2023). The problems that cause deforestation in Indonesia are dynamic, requiring tailored handling at the subnational level and new methods to monitor deforestation ( Austin et al., 2019).

Although it is widely employed, ACP research in Indonesia is currently limited to community and social forestry. Its application outside the forest is still limited, and its innovation and development are still the subject of study by forest policy researchers. Therefore, it is essential to study the ACP approach for reforestation and forest protection in Wallacea to eliminate obstacles related to forest management policies. We take the Wallacea Line as the most biodiverse forest ecosystem where the ACP approach is implemented to highlight how this idea is contested and confronted with the dynamics and more complex societies and ecosystems. We argue that ongoing changes that bias the economic growth or capitalist mode of production have undermined forest protection and rehabilitation in the Wallacea Line. This paper will contribute to the understanding of the ongoing changes due to deforestation in the Wallacea Line, the actors involved, and the proposed policy for better management of forest protection and rehabilitation in the Wallacea region. At the same time, the study also contributes to improving the ACP model itself, which works and is adjusted for diverse and marginal regions.

The paper is organized into six sections. After the introduction, the second section deals with the overview of the Wallacea Line and focuses on the status of the forest and the most degraded ecosystem. The third section discusses the expansion of farming or agricultural land as one of the main contributors to deforestation and land degradation in the Wallacea region. The fourth section discusses the dynamics of land use changes in the Wallacea region. It discusses the development and conservation conducted in two national parks established in the Wallacea region that represent variations of the region’s biophysical characteristics and the actors involved within those landscapes. Section five is a discussion that deals with how the ACP approach can be implemented in the Wallacea region. The last section is a conclusion summarizing the main findings and offering a proposed model for sustainable forest management in the Wallacea region.

## Forest status in the Wallacea line

Forests are an essential resource in the biogeography of the Wallacea ecosystem because they are one of the habitats of plant biodiversity, reaching ±10,000 species; as many as 1,500 plant species are classified as endemic, and 66 plant species are threatened with extinction along with several amphibians, reptiles, birds, mammals, fish, and corals ( Ngakan et al., 2022). Several species are estimated to have not yet been discovered ( Rheindt et al., 2020). The area covered by land and water forests in the Wallacea ecosystem reaches 22,627,571 ha, or 17.99% of Indonesia’s forest area ( BPS Statistics Indonesia, 2021). The forest area is spread across 10 provinces, 134 districts/cities, 1,693 sub-districts, 17,656 villages ( BPS Statistics Indonesia, 2021), and 6,382 islands throughout the Wallacea region ( Liyantono et al., 2022). The region’s population density ranges from 40–290 people/km ^2^ out of a total population of 35,906,794 people ( Ngakan et al., 2022), which will severely impact the pressure on forest resources and land for community livelihoods.

**Figure 1.**
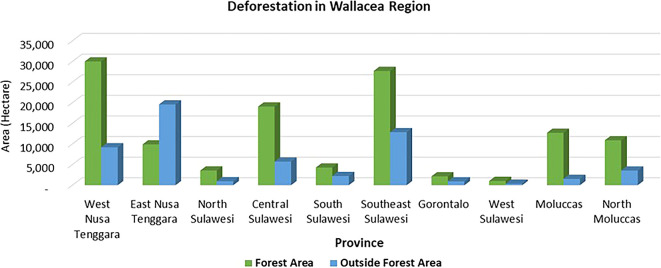
Deforestation in the Wallacea Region, Indonesia, 2017–2020. Source: Processed From BPS Statistics Indonesia, 2021.

Deforestation is a pressing issue in forest management, particularly in the Wallacea region. Most of the deforestation in the Wallacea Line occurred in the last half-century and is closely related to government programs ( Simamora, 2023), agriculture, logging, and mining ( Hance, 2005). The average deforestation rate between 2017 and 2020 was a staggering 163 ha/day or 59,478 ha/year, with 68% occurring in state forest areas and 32% outside state forest areas ( Figure 1). Provinces such as West Nusa Tenggara, Southeast Sulawesi, and Central Sulawesi are witnessing alarming deforestation rates in state forest areas. At the same time, in East Nusa Tenggara areas outside forests dominate. Southeast Sulawesi is experiencing the highest rate of deforestation in both forest areas and outside forest areas. Land fires, a significant factor in deforestation, led to a shocking 29.8% of degraded land in the Wallacea region in 2019 ( BPS-Statistics Indonesia, 2023), primarily due to the long dry season and rampant land clearing through burning. Fire is common in traditional farming practices and bush or grassland management in semi-arid areas of ENT. Indigenous people, particularly in Timor and Sumba, strictly implement Indigenous Knowledge regarding farming to limit the negative aspects of uncontrolled fire. Nevertheless, gradual changes have occurred in traditional fire management, which are most likely related to the introduced crops, livestock, and farming practices that have less considered the semi-arid environment and local people’s practices. In comparing before and current farming practices of Sumbanese, particularly of fire use in farming, Ngongo and Ngongo (2021 p. 4) noticed that the most common practice in fire use before was the use of firebreak and conducted at the end of the dry season or close to the beginning of the rainy season. This traditional practice is less considered in current practice, which leads to wild and uncontrolled fires ( Kapa et al., 2021; Ngongo & Ngongo, 2021).

**Figure 2.**
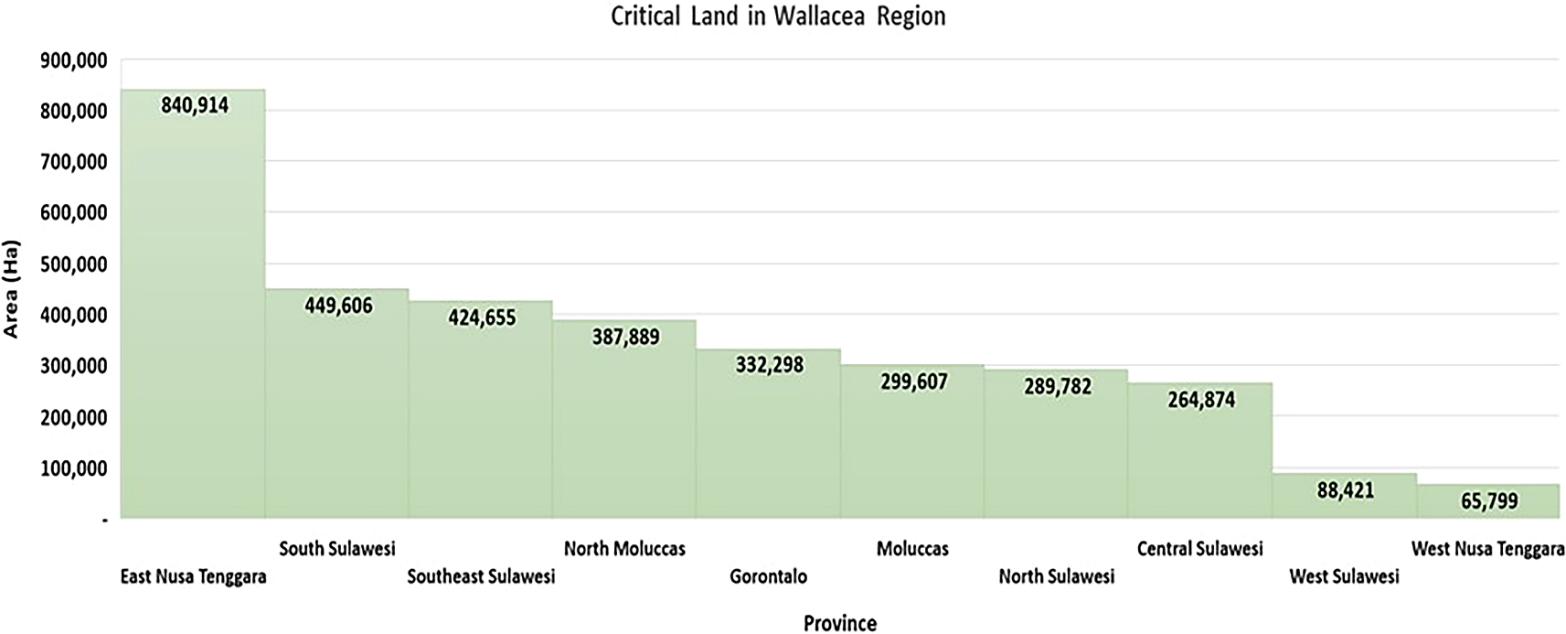
Critical land in the Wallacea Region. Source: Processed From: BPS, 2023.

Pressure on forest areas can be seen from information on the area of critical land in the Wallacea region, which has reached 3,443,845 ha, or 24.59% of Indonesia’s critical land area ( BPS, 2023) ( Figure 2), including pressure on the mangrove ecosystem areas, which cover an area of 354,059 ha in the Wallacea region ( Liyantono et al., 2022). People with livelihoods from the agriculture, forestry, and fisheries sectors spread across 15,874 (89.9%) villages in the Wallacea region face socioeconomic challenges in the use of forest and land resources, including 93.24% of villages being recipients of certificates of incapacity for welfare improvement ( BPS, 2023). It is strongly suspected that socioeconomic vulnerability will affect ecological vulnerability through destructive and environmentally unfriendly land resource use activities. Land fires in the Wallacea region experienced a significant increase in the 2016–2019 period. In 2016, land fires reached 36,980 ha/year, and this increased to 280,835 ha/year in 2019 ( BPS-Statistics Indonesia, 2023).

**Figure 3.**
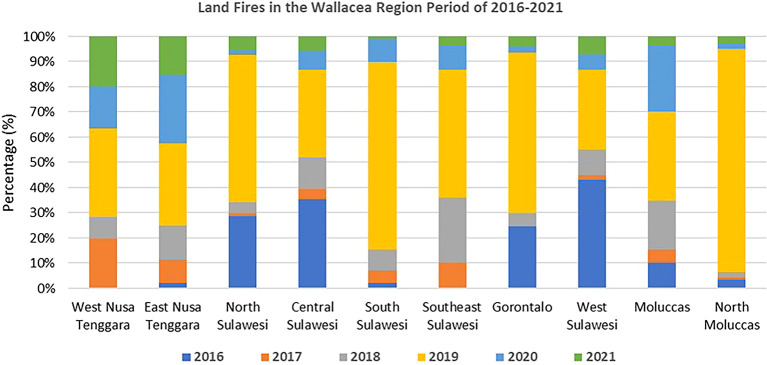
Land fires in the Wallacea Region 2016–2021. Source: Processed From: BPS-Statistics Indonesia, 2023.

Traditional farming practices commonly use fire to prepare land and manage grassland, especially in semi-arid ecosystems in East Nusa Tenggara ( Ngongo et al., 2021, 2022; Tacconi & Ruchiat, 2006). Expansion of agricultural land and deforestation contributed to the increases in fire, land degradation, and air pollution in Sulawesi ( Amin et al., 2022; Supriatna et al., 2020). Climate and protracted fires in Wallacea have contributed to the ecosystem dynamics ( Hamilton et al., 2019). However, fast changes in land use, particularly for large-scale oil palm expansion, have caused frequent fires on a large scale ( Figure 3) that threaten the region’s biodiversity ( Liyantono et al., 2022).

East Nusa Tenggara Province has a critical land area of 840,914 ha, the highest amount of all provinces in the Wallacea region. Some of the causes are: (a) shifting cultivation activities characterized by burning weeds and grass with a short rotation period have implications for the process of restoring land fertility, (b) wild grazing is still the dominant choice because the number of livestock and labor capacity for grazing has not been balanced, which encourages the burning of pastures, negatively affecting the chain of regeneration of seedlings into trees, (c) slash-and-burn agriculture continues to strengthen as a result of tradition and poverty, for energy substitution and low-cost labor, and (d) the use of fire for the transformation of energy and nutrient inputs in the farming system is still applied in 2,306 villages around forests in East Nusa Tenggara (ENT) ( Njurumana, Riwu Kaho, et al., 2021b), and (e) there is still a gap between the level of awareness and the sporadic implementation of soil and water conservation, with situational and limited adoption of technology. The practice of shifting cultivation with slash-and-burn systems is still common and has implications for the increase in degraded land ( Abrell et al., 2024; Baul et al., 2023), driving forest destruction and increased erosion ( Wang et al., 2023), as well as other environmental problems, such as biodiversity damage and climate change ( Hazarika et al., 2024; Leddin, 2024; Weiskopf et al., 2024).

## Agriculture and deforestation in the Wallacea Line

Externalities are processes that can cause benefits or losses to others ( Grafton & Flanagan, 1995). They are side effects of economic activity ( Helbling, 2010). Helbling (2010) emphasized non-excludable externality when the calculation between negative and positive effects becomes blurred or unrealistic in general goods. Increases in crop production and economic gains from timber production on one side have brought negative environmental consequences on the other side: land degradation, biodiversity loss, and negative sustainable development ( Damania et al. 2018). The potential of forestland that can be developed for agriculture in Indonesia is around 50.19%, included in the categories of Other Land Use Areas (OLUA), Limited Production Forest (LPF), and Production Forest (PF) ( Ritung et al., 2015). Thus, the potential for deforestation and forest degradation is alarming, as land demand increases with negative external consequences. Forest displacement generates positive externalities, such as climate regulation, biodiversity conservation, carbon storage, erosion control, and non-timber values ( Maertens et al., 2022).

Environmental externalities are problems that cannot be separated from economic activities carried out by the community. Deforestation and forest degradation negatively impact health, the environment, and economic development. Forests are negatively impacted by economic activity. Deforestation in Wallacea is accompanied by increasing forest fragmentation. The fragmentation of forest ecosystems has far-reaching impacts on biodiversity, degrading key ecological processes and altering nutrient cycles ( Haddad et al., 2015). Deforestation and degradation of the Wallacea hotspot have reduced the number of forest habitats, especially in the lowlands, and have led to dramatic and severe declines in the populations of many forest species ( World Bank, 2011). Over half of the country’s species are on the IUCN Red List ( Supriatna et al., 2020).

Indonesia has the fastest annual deforestation rate in the world, with 1.8 million ha of forest being destroyed yearly between 2000 and 2005. The rate of forest destruction is 2% every year, which is equivalent to 51 km ^2^ per day ( Arif, 2016). Deforestation is the condition of forest areas that have decreased due to land conventions for infrastructure, settlements, agriculture, mining, and plantations ( Yakin, 2017; Arif, 2016). Large parts of Wallacea, particularly Sulawesi and Nusa Tenggara, have lost substantive forest cover due to urbanization, agricultural land, and mining ( Budiharta et al., 2018).

Sulawesi, part of the Wallacea hotspot, has seen deforestation and forest degradation since the early 1970s, when forests were cleared for agriculture through transmigration programs ( Whitten et al., 1987). In the early 1980s, gold and nickel mines were opened, while oil plantations were established in the early 1990s in West Sulawesi Province and later in Gorontalo Province. Forests lost from the western part of West Sulawesi have been converted to oil palm farmland.

In Southeast Sulawesi, forestland is cleared and used for mixed agriculture, including oil palm, maize, and cocoa. The expansion of oil palm plantations that cause deforestation impacts local communities’ environmental and economic problems. There have been many empirical studies in various places related to the impact of oil palm plantation development on environmental damage ( Susanti & Maryudi, 2016). The expansion of oil palm plantations has increased carbon emissions, and climate change can disrupt environmental conditions ( Pacheco, 2012). Nickel and gold account for most of the mining output in Sulawesi. Nickel is found in Central, South, and Southeast Sulawesi. Central and Southeast Sulawesi, in particular, are affected by large mining operations, with final products being exported to China. ( Song et al., 2018). According to Damania et al. (2018), road infrastructure for mining operations can cause deforestation and biodiversity loss.

There was a decline in forest cover in Sulawesi Island of 2,069,016.66 ha, or 10.89%, between 2000 and 2017 ( Supriatna et al., 2020). Forest cover loss includes protected areas and national parks. The most extensive forest loss, 737,516.52 ha, was in Central Sulawesi Province in the last three decades (1990-2020). Average deforestation in Sulawesi ranges from 0.42% to 0.85% annually, with the main drivers for expansion of agricultural land and timber production ( Rijal et al., 2019; Supriatna et al., 2020; Syah et al., 2018). The highest rate of deforestation is found in Southeast Sulawesi Province, and reaches 0.85% each year. Rijal et al. (2019) assessed more significant forest loss in Sulawesi at 18.90% from 1990 to 2018, when most of Sulawesi’s lowlands were cleared for agriculture.

Deforestation is due to the expansion of agricultural land in Sulawesi, where 1.4 million hectares of land are suitable for use as rice fields. Most of this (covering an area of 1.1 million hectares) is non-swamp/tidal land. In the medium term, a realistic assumption for agricultural land expansion in Sulawesi is 15%. Thus, the potential for expanding paddy fields is around 200 thousand hectares. Suppose it is assumed that the potential is around 10%. In that case, the total land area that can be considered potentially used for dryland expansion in the medium term is around 1.6 million hectares. Its spatial distribution in Sulawesi covers an area of 205 thousand hectares ( Bappenas, 2010).

Furthermore, the availability of land in Sulawesi that is suitable for the expansion of rice fields is 422,972 ha, spread across the provinces as follows: North Sulawesi, 26,367 ha; Gorontalo, 20,257 ha; Central Sulawesi, 191,825 ha; South Sulawesi and West Sulawesi, 63,403 ha; and Southeast Sulawesi, 121,122 ha. These lands are generally located on alluvial plain landforms, river flow paths, and hinterland plains, whose soil develops from alluvium deposits ( Ritung & Las, 2009).

Indonesia’s population continues to grow; the population in 1970 was 115.22 million people; in 1990, it increased to 182.160 million, and in 2022, it became 275.501 million ( Wordometer, 2024). This population growth has led to a rise in the need for clothing, shelter, and food, as well as to an increase in the migration process. This increased need for clothing, shelter, and food production must be balanced with the expansion of new areas or extension of agricultural land, which can be done through forest clearing.

The forest area in Indonesia in 2020 was 120.2 million ha, while the determined forest area was ± 88.4 million ha. Although deforestation in Indonesia is still ongoing, the rate of deforestation declined to 75% between 2019 and 2020, or around 115,5000 ha, compared to 462,500 ha between 2018 and 2019 and 480,000 ha between 2017 and 2018 ( Liyantono et al., 2022). The historic low deforestation in 2020 was attributed to government policies prohibiting forest clearing, more rains, falling palm oil prices, and the economic downturn due to the COVID-19 pandemic ( Jong, 2021).

## Cocoa discourse: Between deforestation and new livelihood

Cocoa is one of the leading export commodities for the Indonesian economy, which contributes 3.94% to Indonesia’s GDP ( Statistics Indonesia, 2022). Sulawesi, an island of Indonesia, is the largest national cocoa producer with a production of 403.6 thousand tons ( Statistics Indonesia, 2022). Unfortunately, the expansion of cocoa plantations has led to a significant increase in land clearing, particularly of forested areas, resulting in deforestation and broader environmental problems. Farmers often clear land by cutting down forests to make room for cocoa crops, as the fertile soil in the forest area provides higher yields.

The rate of deforestation in Sulawesi for the period from 1990 to 2018 reached 18.9% ( Rijal et al. 2019), which devastated more of the lowland areas that are suitable for plantations and other agricultural products ( Supriatna et al., 2020), and even people encroached on forest-protected areas ( Kalischek et al., 2023; Parra-Paitan et al., 2024). Cocoa-driven deforestation causes various problems, such as the threat of biodiversity loss, erosion, land degradation, and disruption of hydrological functions ( Kouadio & Singh, 2021), threatens biodiversity, and affects ecosystem functions ( Kamath et al., 2024).

Critics of the cocoa plantations’ expansions in Sulawesi seem more focused on land conversions -mostly forest lands- which lead to deforestation, biodiversity loss, and disturb the ecosystem; however, there is potential to improve this situation through a sustainable agricultural approach. Adopting an agroforestry system, in which cocoa plants are planted along with forest trees, can help maintain forest cover while still supporting cocoa productivity. The development of cocoa-based agroforestry is one of the important strategies to overcome agricultural land shortage as the human population increases ( Murniati et al., 2022). Furthermore, cocoa grown in the shade of forest trees can result in more production ( Isaac et al., 2015). Therefore, by adopting proper cultivation or environmentally sound technology, cocoa farming is no longer relevant as a cause of deforestation.

The cocoa farming supports poverty alleviation for rural people since it is operated mainly by smallholders, receives less government support, and uses fewer external inputs ( Ruf et al., 1996). Drawing from ethnographic research on the island of Sulawesi, Indonesia, Li (2023) noticed that farmers’ responses to embrace the Cacao innovation as a new livelihood opportunity. She then challenges the dualism theory and rejects the myth of the lazy native.

## Changes in land use and biodiversity

**Figure 4.**
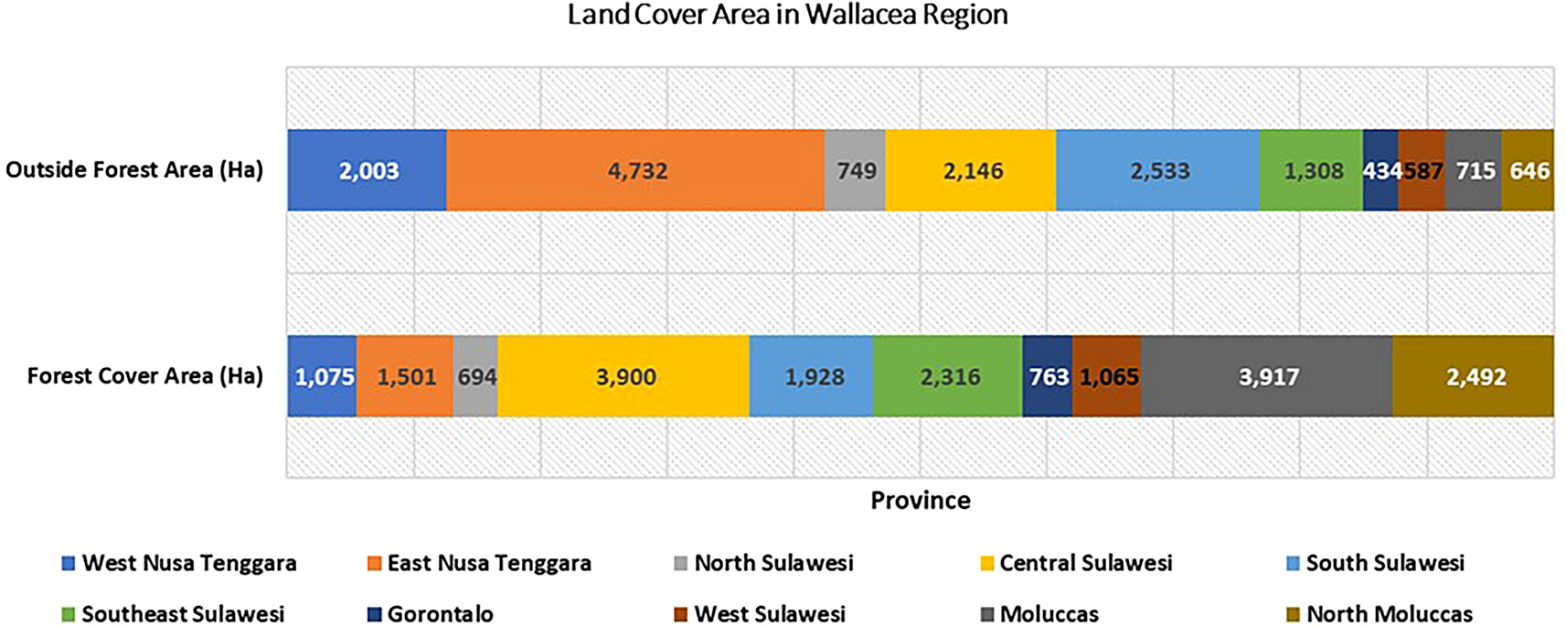
Land cover area in Wallacea Region (.000 ha). Source: Processed From: BPS-Statistics Indonesia, 2023.

Changes in land use have implications for the biodiversity of flora and fauna and their ecosystem services. Population growth has implications for increasing land pressure, especially the dominance of people who depend on the agricultural sector and the use of agricultural practices that are not yet environmentally friendly. This will affect the increasing land pressure on the Wallacea ecosystem. In analyzing land use change and biodiversity, it is necessary to understand the condition of land cover within and outside forest areas ( Figure 4). We found variations in the dominance of land cover in Wallacea forest areas from Maluku, Central Sulawesi, Southeast Sulawesi, and East Nusa Tenggara. Conversely, the dominance of forested land cover outside forest areas from East Nusa Tenggara, South Sulawesi, West Nusa Tenggara, and Southeast Sulawesi is thought to be the impact of community forest expansion by the community. This indicates that parties’ participation in forest management has begun to increase, and community forest units in various forms have developed to form a canopy stratification that resembles natural forests.

The Wallacea region is known worldwide as a meeting point for the biogeography of Asia and that of Australia ( Struebig et al., 2022). Generally, the Wallacea region’s islands can be divided into three groups, namely Sulawesi, Lesser Sunda, and Moluccas ( Yuni & Yuda, 2019). The islands in the Wallacea region have the highest level of endemicity in the world ( Struebig et al., 2022), with 57% of the mammal species, 40% of the bird species, 44% of the reptile species, 65% of the amphibians, and 20% of the freshwater fish species found in this area being endemic ( Yuni & Yuda, 2019).

The largest island in the Wallacea region ( Ono et al., 2022), Sulawesi Island also has the largest forest cover. The forest area on Sulawesi Island covers 56% of the Wallacea region, while the forest area on the Maluku Islands covers 24%, and that of the Lesser Sunda Islands only cover 19% ( Yuni & Yuda, 2019).

The Wallacea bioregion has a variety of forest types. On Sulawesi Island, for example, you can find mangrove forests, coastal forests, swamp forests, lowland forests, lower mountain forests, upper mountain forests, and seasonal forests, including forests that grow on volcanoes and limestone ( Whitten et al., 1987). These various types of forests provide a variety of habitats and, at the same time, various types of endemic fauna, especially mammals and birds ( Yuni & Yuda, 2019). The Maluku Islands have quite a few forest types, such as mangrove forests ( Salampessy et al., 2024), coastal forests ( Pugesehan, 2009), lowland forests ( Basri & Yuniarti, 2019), lower mountain forests ( Mirmanto, 2010), higher mountain forests ( Mirmanto, 2010), forests on limestone ( Roemantyo, 2017), dryland forests, and savannas ( Parera et al., 2022). Meanwhile, on the Lesser Sunda Islands, you can find peat swamp forests, lowland rain forests, lowland monsoon forests, forests on limestone rocks, forests on ultrabasic rocks, tropical montane forests, savannas, and grassland ( Monk et al., 1997).

Unfortunately, forest areas in the Wallacea region are under high pressure due to increasing populations and various community activities, with community life and economic growth relying on the exploitation of natural resources ( Struebig et al., 2022). Based on the deforestation model created by Voigt et al. (2021), there was a decrease in forest area of more than 10,231 km ^2^ from 2000 to 2018. The decrease in forest area is estimated to increase to 49,570 km ^2^ in 2053 ( Voigt et al., 2021). This has caused the Wallacea bioregion to become an area with a high level of biodiversity threat because half of the types of Indonesian biodiversity that are included in the IUCN Red List are species that live in the Wallacea bioregion ( Supriatna et al., 2020; Voigt et al., 2021). In the Sulawesi region, changes in forests and land use began to increase rapidly around the 1970s, when the government began carrying out forest logging, transmigration, and plantation projects. Central Sulawesi was listed as one of the main timber producers in Sulawesi in the 1970s. This has caused forests in Sulawesi to only be found in the form of spots, which are often separated from one forest area to another ( Whitten et al., 1987).

The rapid deforestation and land use change in the Wallacea bioregion led to forest areas only being found in conservation areas or in steep locations and areas unsuitable for agriculture, plantation, mining, and other changes. Forest loss occurred not only on the main island of Sulawesi but also on small islands. Several small islands have lost their natural forests and turned into agricultural regions, such as Sangihe, Selayar, and Kalaotoa ( Whitten et al., 1987). The following subsections mention the three forest sites representing the main islands with higher rainfall (Sulawesi—tropical rainforest) and lower rainfall (Timor and Sumba islands—dryland/semi-arid forests). However, land cover change mapping has only been analyzed for Mount Mutis and Manupeu Tanadaru Laiwanggi Wanggameti (Matalawa).

Land cover change was researched to identify the classification and analyze land cover changes in the Mount Matalawa areas using the Geographic Information System (GIS). Map processing was done using the Qgis (Open source) application. Processing was done by changing the position/coordinates of Landsat 5 TM and 8 OLI data by WGS 1984 UTM Zone 51 S to get a natural position from the research area. Land cover classification is based on land use type and heading density, such as dryland agriculture, mixed dryland agriculture, natural forests, secondary dryland forests, shrubs, and rice fields. The Landsat Imagery data for 2000 and 2020 consisted of Landsat 5 TM, Landsat 7 ETM+, and Landsat 8 ETM+. The multitemporal data analysis process involves three stages: preprocessing, processing, and image classification. This method identifies changes in land cover in the forest ecosystems of Mount Mutis and Matalawa.

### Mutis forest of Timor Island

**Figure 5.**
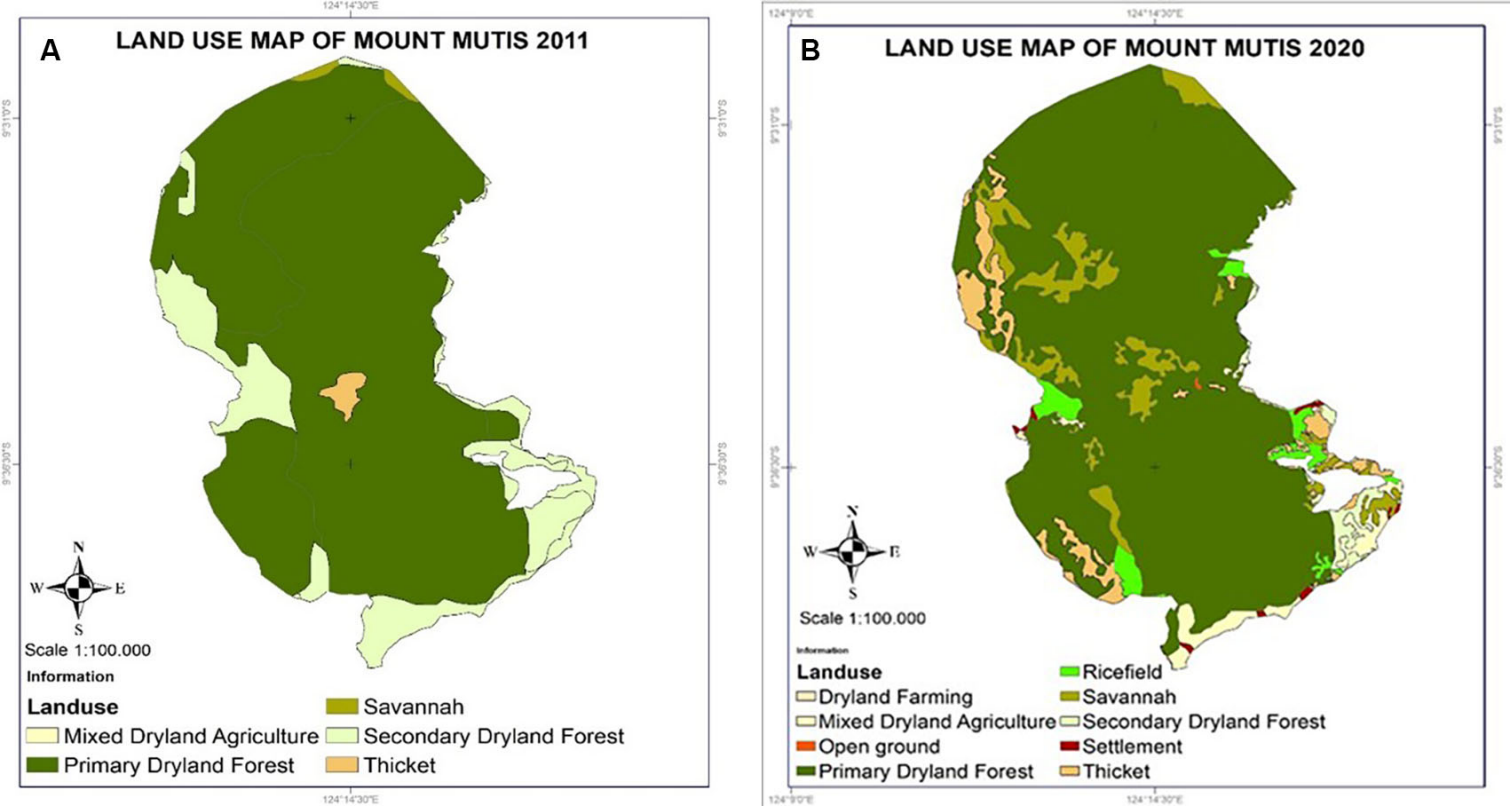
Land use changes in Mount Mutis, West Timor.

The Mutis forest area, covering an area of 31,917 ha ( Figure 5), is one of the mountain forest ecosystems of strategic value ecologically, socioculturally, and economically on the island of Timor, NTT ( Budiman et al., 2020; Dako et al., 2018; Pujiono et al., 2019). Ecologically, the Mutis forest area consists of 19,586 ha of protected forest and 12,315.61 ha of nature reserves ( Njurumana, Riwu Kaho et al., 2021b), and it is one of the most mountainous tropical forest landscapes in Indonesia ( Pujiono et al., 2019). The Mutis forest area plays an important role as the heart of the catchment area defense for three main watersheds on the island of Timor, namely the Benain watershed, the Noelmina watershed, and the Noelfail watershed ( Njurumana, Riwu Kaho et al., 2021b). The three watersheds cross five of the six districts in West Timor, including a small part of the Republic Democratic of Timor Leste (RDTL) ( Dako et al., 2018; Riwu Kaho et al., 2020).

The Mutis forest area is a source of livelihood for at least 4,643 families, so deforestation pressure due to dependence on firewood reaches 87.45% /year; carpentry wood reaches 97% / (15–20 years) ( Dako et al., 2018); and livestock is raised illegally ( Mulyadi et al., 2022). Poverty is one factor that increases dependence on forests, especially in underdeveloped and poor villages ( Njurumana, Pujiono et al., 2021a), with food expenditures of Rp. 398,450–Rp. 609,150/capita/month ( BPS-Statistics Indonesia, 2023). Conditions differ, with forest farmers’ income ranging from Rp. 201,900–368,900/capita/month ( Dako et al., 2018; Njurumana et al., 2020), lower than the per capita expenditure at the South-Central Timor Rp. 447,600–Rp. 1,602,250 ( BPS TTS, 2022). Several villages around the Mutis forest area include Kuanoel, Fatumnasi, Nenas, Nuapin, Tutem, Tune, Bonleu, Noepesu, Fatuneno, Tasinifu, and Fatukoto.

There has been a decline in Mutis forest ecosystem services. This is illustrated by, for example, honey production dropping to 53.12% of the average production for a decade ( Njurumana, Riwu Kaho et al., 2021b). We suspect that this is due to the decline in the habitat for honeybees, hive trees, and pollination due to pressure that has caused a decrease in densely vegetated forests in the last 30 years in the Mutis forest ( Pujiono et al., 2019). This change can affect the carrying capacity for water conservation for the micro-hydropower plant, bottled water, and regional water supply companies.

Local communities are one of the determining actors in forest sustainability; for a long time, they have interacted with various forest functions for protection or conservation, production, sociocultural (customary), and protected forests. The sociocultural and ecological relations between the community and the Mutis forest are very strong, acting as *Faut-Kanaf Oe-Kanaf* (Batu Nama, Air Nama), which is believed to have historical value as a cultural-humanist identity of the Dawan ethnic ancestors and their descendants as well as spiritual (sacred) values as the center of human relations nodes with *Uis-Neno* and *Uis-Pah* (ruler of the sky and ruler of the earth). Uis-Neno is described as *Apinat ma Aklaat* (true sun and light), *Amoet ma Apakaet* (ruler of heaven and earth), and *Alikin ma Apean* (giver of life to all beings). *Uis-Pah* is the ruling spirit of the universe (earth) that dwells in certain spaces (trees, rocks, rivers, mountains) and is used as a sacred place by the Dawan ethnic to carry out rituals periodically.

Relevant to sociocultural values in the use of forests and their ecosystems, the Mutis people apply the *mansian muit-nasi nabua* philosophy, the concept of the triangle of life that harmonizes the relationship between humans, forests, and livestock as living units that are inseparable from each other ( Njurumana, Pujiono et al., 2021a). Humans manage forests by adhering to historical and sociocultural values, thus placing Mutis as one of the centers of Dawan ethnic civilization. Several *Suf areas*, namely traditional/customary management areas practicing customary law, are evidence of the existence and manifestation of sociocultural values in Mutis forest management ( Budiman et al., 2020; Mulyadi et al., 2022; Njurumana, Pujiono et al., 2021a). On the other hand, the Mutis forest provides benefits for human livelihoods, such as food, firewood, and forest honey ( Dako et al., 2018; Mulyadi et al., 2022; Njurumana, Pujiono et al., 2021a), and the long-standing application of silvopasture in the Mutis forest area is part of the livelihood and social prestige of local communities ( Ngongo et al., 2022). This philosophy encourages local communities to have the obligation to maintain forests, keep the sacredness of customary prohibition areas, use animals sustainably, and maintain and obey customary rules governing forest protection, including maintaining social-kinship relations between traditional leaders and communities.

The early introduced forage legume tree species in the semi-arid region of East Nusa Tenggara (ENT) was Leucane leucocephala. Leucaena was introduced by the Dutch in the early 1930s to overcome the invasive species of *Lantana camara*, improve land conservation, and soil fertility in semi-arid areas ( Ormeling, 1957; Fox, 1988). The introduction of Leucaena started in Timor and then spread to other regions of ENT. Later, it became a pivotal innovation transforming traditional farming and livestock raising systems, particularly in the Amarasi region of Timor. The current study by Ngongo et al. (2021, 2022) In Timor, the Introduction of L. leucocephala has contributed significantly to the decrease in shifting cultivation, less use of or no fire in land preparation, improving crop production, especially maize, cattle keep gaining weight even in the long dry season, and increasing the number of cattle fattened per household (p. 5). Leucaena in Sumba is mainly used for shading three and nitrogen-fixing trees in a traditional agroforestry system. Like Sumba, Leucaena in Flores controls soil erosion and is used for shade trees, particularly cocoa and coffee trees.

### Manupeu-Tanadaru Laiwanggi-Wanggameti (MATALAWA) in Sumba Island

Sumba Island experiences ecological vulnerability due to land cover change, which tends to decrease due to land degradation reaching 6000 ha/year, so that forest cover is only ±7% ( Sitompul et al., 2004), spread over several forest blocks with a wide range of 16–42,500 ha. This causes the land cover of Sumba Island to be dominated by shrubs and savannas. Shrubs in forest areas reached 57,811 ha or 15.41%, while savannas reached 169,340 ha or 45.14% ( Balai Taman Nasional MATALAWA, 2020) ( Figure 3).

**Figure 6.**
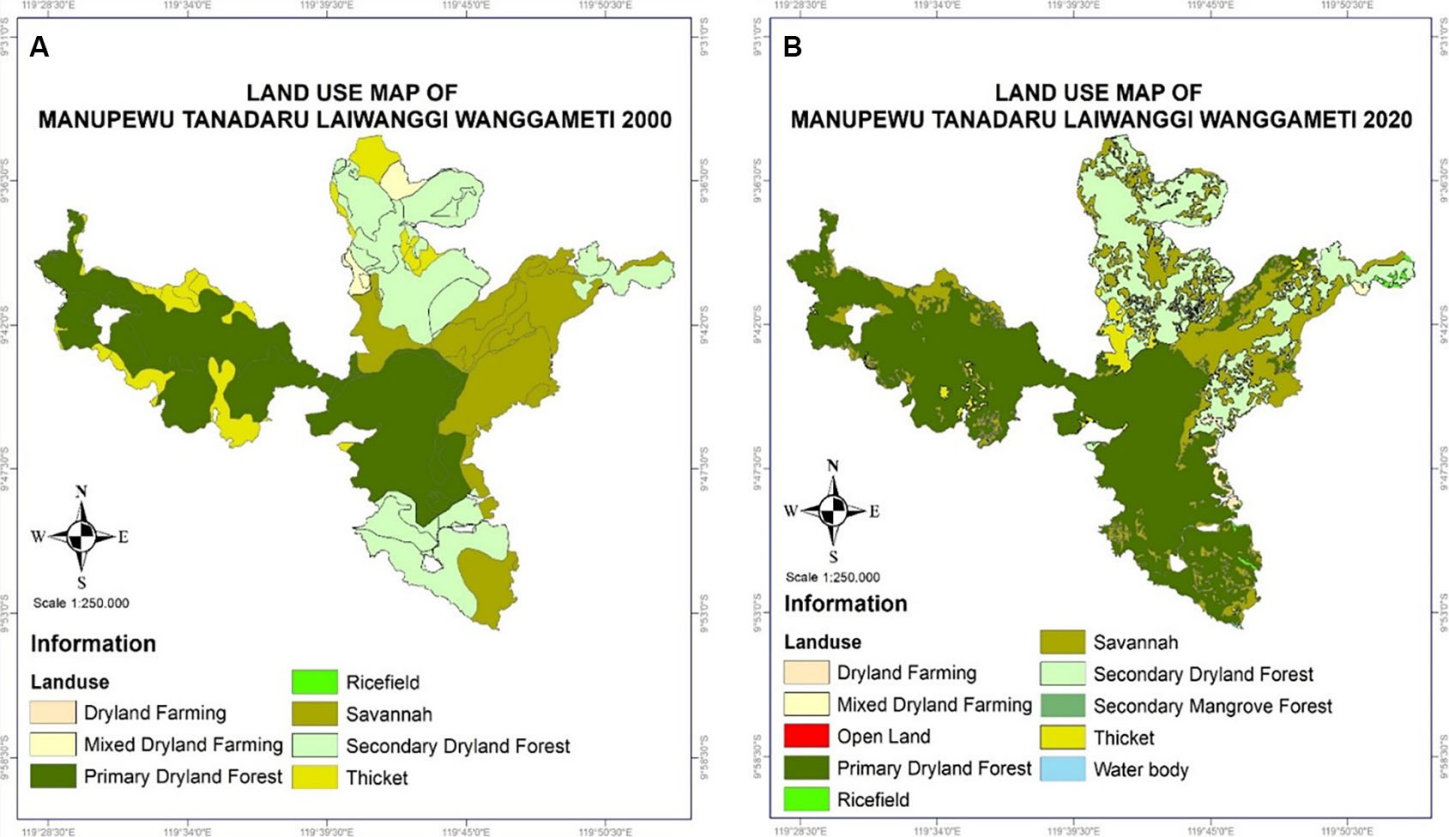
Land use change in Matalawa National Park, Sumba Island.

Manupeu Tanah Daru and Laiwangi Wanggameti National Park (Matalawa) ( Figure 6), which was established by Regulation of the Minister of Environment and Forestry of the Republic of Indonesia Number P.7/Menlhk/Setjen/OTL.0/1/2016 concerning the organization of Matalawa National Park, covers an area of 134,998.09 ha across three districts: West Sumba, Central Sumba, and East Sumba. Natural forest-type formations range from coastal forests to lowland semi-green forests and from savannas to the dominant dry tropical forests, which are habitats for the endemic birds of Sumba ( Wibawanto, 2011). One of the typical forest ecosystems in Sumba is the tropical elfin forest and Sumba deciduous forest, while the type of elfin ecosystem and monsoon forest ecosystem is unique and has not been found in other regions of Indonesia ( Hamidi et al., 2017).

Fauna biodiversity, especially birds, includes the endemic species of Sumba and migrant species, reaching 159 species. Some endemic species are flagship Matalawa species, such as *Cinnyris buettikoferi*; *Dicaeum wilhelminae*; *Myzomela dammermani*; *Turnix everetti*; *Treron teysmanii*; *Ptilinopus dohertyl*; *Ficedula harterti*; *Muscicapa segregate*; *Ninox sumbaensis* and *Ninox rudolfi*; *Eclectus roratus cornelia*, including *Rhyticeros everetti*; and *Cacatua sulphurea citrinocristata* ( Yusuf et al., 2017) *.*

Disruption to forest management has occurred in logging, encroachment, and hunting. In addition, land fires reached 533,881 ha, triggered by livestock grazing, opening agricultural land, long droughts, and limited accessibility to risk mitigation ( Balai Taman Nasional MATALAWA, 2020). The establishment of new districts and expansion of agricultural land, particularly for sugarcane and rice estates, have also contributed to changes in land use on Sumba Island ( Ngongo et al., 2023). The pressure on forest areas cannot be separated from the existence of communities around the forest that need access to forests to extract building wood and firewood and to hunt for non-timber forest products, including 164 types of medicinal plants in the national park, such as *Imperata cylindrica*, *Curcuma longa*, and *Grewia acuminata* ( Kusumanegara et al., 2020). Underprivileged communities in buffer villages around Matalawa have low incomes, resulting in high dependence on forest resources ( Balai Taman Nasional Matalawa, 2019), and there are at least 55 buffer villages, most of which are in East Sumba Regency.

The increase in land cover in Matalawa National Park in the last 20 years is suspected to be the result of two main factors: (1) the success of managers in mitigating land and forest fires, controlling illegal logging, and increasing counseling and conservation partnerships and local community participation in forest protection; (2) the success of the community in managing Kaliwu—local agroforestry—as a replication of the natural forest model in the farming environment with various benefits, including: (a) contributing to the conservation of plant biodiversity, reaching 145 species ( Njurumana et al., 2016); (b) contributing to building timber ranging from 59%–96% ( Njurumana et al., 2016); (c) contributing to household income ±46% ( Njurumana et al., 2016), (d) contributing to food in the range of 39%–41%; and (e) contributing to fruits in the range of 66%–69% ( Njurumana, Sadono, et al., 2021c).

### Reforestation on the Main Island of Sulawesi

Sulawesi is the largest island, covering more than half of the terrestrial and marine habitat in the Wallacea region. The subregion of Sulawesi, and its surroundings, is rich in biodiversity; however, these species are mostly on the IUCN’s Red List ( Feagan, 2017). Therefore, the conservation of natural resources and the ecosystem should be conducted using an integrative approach for appropriate results ( Cannon et al., 2007), particularly in Sulawesi terrestrial and marine ecosystems, as this will very much contribute to ensuring the conservation of endemic species in the Wallacea region ( Struebig et al., 2022).

Reforestation is a movement to reforest forests that have been deforested so that they can function properly ( Southworth & Nagendra, 2009). Reforestation is one of the efforts made in dealing with environmental damage, so reforestation activities involve many parties/actors in the use of resources, including the community ( Innah et al., 2013). Forest and land degradation in South Sulawesi province has been indicated by increased greenhouse gas emissions, mainly from the land sector, which showed that greenhouse gas inventory (CO _2_) emissions from the land sector amounted to 2,057 Gg in 2015, an increase of 1,933 Gg when compared to 2009, when the level was only 124 Gg. Forest and land degradation is generally caused by the increased conversion of forestland for the plantation activities of communities living around forests as an economic measure to increase income and meet family needs. In addition, illegal logging and exploitation practices are also carried out by entrepreneurs who obtain HPH/IUPHHK permits. Logging is carried out on production forestland, in protected forests, and in conservation areas, including national parks and wildlife reserves ( Badan Lingkungan Hidup Daerah Sulawesi Selatan, 2016).

Environmental efforts sometimes clash with various interests. This happens because the need for land continues to increase as a medium for forestry, agriculture, and plantations, and this can cause conflicts between the actors involved. The Indonesian government initiated the resolution of agrarian conflicts through the TORA and Social Forestry programs. However, the program involves various actors in its governance, which can potentially affect its success. Bimantara’s (2022) observations concluded that the TORA program uses power relations analysis by looking at the patronization of the actors.

Three bureaucracies are the most influential patrons and/or actors and have a vital role in the implementation process in South Sulawesi Province. The bureaucratic patronage includes (a) the South Sulawesi Provincial Forestry Service, (b) the Regional Office of ATR/BPN South Sulawesi Province, and (c) the Forest Area Designation Bureau (BPKH). As for Social Forestry (SF), influential actors are the South Sulawesi Provincial Forestry Service, the Watershed Management Center (BPDAS), and the Sulawesi Social Forestry and Environmental Partnership Center (BPSKL). Hasanuddin University Institution owns the power legitimacy (PL) category, and RECOFTC, CIFOR, and AgFor own the power interest (PI) category. Forestry extension officers own the interest legitimacy (IL) category. Category I (interest) is owned by SCF, Balang, Kareso, Lampion, Yagrobitama, Walda, TLKM, and AMAN. The management of the TORA certification presents actors who become brokers, while SF approval involves many actors, so several miscommunications impact conflicts between actors due to differences in interests. This study shows that the involvement of actors and the existence of conflicts of interest affect the implementation and agenda of government programs.

Regarding reforestation efforts through the SF program, which uses land cover indications, Sahide et al. (2020a) stated that 42.8% of SF forest farmer groups in South Sulawesi have not made a tangible contribution to increasing land cover outside forest areas or the work areas of farmer groups. Some things that are indicated to affect the case include forest conservation actions that have not been listed in the work plan document of the SF group, forest utilization business permits being issued in a span of less than one year, and the fact that several social forestry areas have a very high land density in the form of existing trees so that no more land can be planted with trees.

Forest rehabilitation is an effort to plant forest tree species in damaged forest areas in the form of vacant land, reeds, or shrubs to restore forest function. Reforestation is prioritized in protected forest areas, which aim to restore the main function of protection of life support systems to regulate water management, prevent flooding, control erosion, prevent seawater intrusion, and maintain soil fertility. Land rehabilitation activities aim to plant trees (conduct reforestation) on critical land outside forest areas ( MoEF, 2021). One of the challenges in forest and land rehabilitation in Indonesia is the economic interest involving various actors. Therefore, the success of land rehabilitation depends on stakeholder synergy between communities, private sector actors, and the government ( Liyantono et al., 2022).

To support forest and land rehabilitation activities and the provision of productive seeds for the community, the Indonesian government, in collaboration with state-owned enterprises and the private sector, has facilitated tree seedlings sourced from 57 permanent nurseries being spread throughout Indonesia, including in Likupang, North Sulawesi Province. This policy has contributed to improving the forest cover on Sulawesi Island, including in South Sulawesi Province, which in 2022 recorded a forest cover of around 1.5 million hectares or 32% of the total land area ( Amin et al., 2022).

South Sulawesi has at least three types of ecosystems that are very rich in biodiversity: (1) highland-mountain ecosystem, (2) lowland-inland ecosystem, and (3) coastal and marine ecosystem. In the two types of ecosystems mentioned first, no less than 64 species of fauna and 149 species of flora are protected. Changes in land use existed across districts in South Sulawesi, and the lowest forest cover of fewer than 1,000 hectares existed in the four districts of Makassar, Jeneponto, Pare-pare, Takalar, and Wajo, while the highest forest cover area from 350,000 to less than 500,000 hectares existed only in two districts, East Luwu and North Luwu ( Amin et al., 2022). The changes in land use that tend to decrease forest cover have huge implications for the region’s biodiversity. Some forest vegetation species have been in danger and are therefore under protection, such as 45 species of trees, nine species of palms and ferns, 95 species of Orchidaceae, and three species of Nephentanceae ( Amin et al., 2022).

## Discussion: Actors in Community Forest Management in Wallacea Region

### Identified actors

Community forest management in the Wallacea region cannot be separated from the role of the actors in it. Some are key actors from the provincial and district levels, such as provincial and district forest services. Other main actors are farmers, farmer groups, timber traders, brokers, sawmills, and boat industries. The secondary actors are the village governments, industry office, food crops office, livestock office, and community and village empowerment office. Almost all actors are formal actors and or government-induced actors. In contrast, non-formal actors and/or grass-roots actors are less represented and less involved in the decision-making process for forest management.

We took the Bulukumba District of South Sulawesi Province for a deeper understanding of the actors involved in forest management by considering the existence of deforestation, reforestation, and previous studies conducted. The Forestry Service of Bulukumba District of South Sulawesi Province is a key actor in community forest service at the district level. The Forestry Service of Bulukumba District influences other actors in two ways: disincentives/incentives and information dominance. Meanwhile, coercion cannot be applied to community forests because forestland is under the authority of landowners. In contrast, state forests require coercion in their management because the security and protection of state forests in involved, where the government as a formal actor exercises its power through coercion ( Charmakar et al., 2024).

Regarding community forest products such as timber, the government cannot play a role in determining prices. The prices of products from community forests, especially timber, follow market mechanisms. In addition, the capital used does not come from government assistance. There is a difference in prices determined by the timber industry and traders, which is related to the operational costs of timber harvesting. Higher prices are offered by the timber industry compared to timber traders. The high price offered by the industry is due to the cost of harvesting and transporting to the industry, which is borne by farmers. Meanwhile, if farmers sell to timber dealers, the costs are borne by traders. In certain cases, farmers cannot control the price of wood because the supply of wood is greater than the demand (Shah et al., 2018).

Disincentives are imposed in terms of receiving assistance intended for smallholder forest farmers. Farmers who lack capital need production facilities and infrastructure. They are directed to form farmer groups if they want to improve facilities and infrastructure. This is intended to assist monitoring, evaluation, control, and distribution. The government’s incentives include seeds, fertilizer assistance, and land processing. Meanwhile, the information provided is about the economic potential of the standing plants.

To improve the local economy through community forest management for timber supply and to address environmental issues, the Forestry Service of Bulukumba District incentivizes community forest farmers. The incentives provided include facilitating infrastructure facilities from upstream to downstream, providing regulatory support, and providing capacity building (training) in technical and marketing aspects. In this case, farmers, as the recipients of these incentives, do not have other more profitable options; however, if there were more profitable incentives, farmers would not follow the wishes of the government ( Krott et al., 2014). The community depends on and is affected by the incentives offered by the government (Environment and Forestry office) to plant forest plants on their land.

Numerous traditional actors are involved in forest and land resource management in Wallacea regions; however, their existence and functionality are closely related to the specific ethnic and culture. The Tobe traditional institution of the Meto tribe in Timor, for example, is responsible for managing land forest resources, including sandalwood ( *Santalum album*) management ( Ngongo, 2011). The current conditions of the Tobe are no longer functioning, and they have been replaced with more induced formal institutions such as farmers’ groups, village forest management groups, or village business groups in managing forest resources.

Local or traditional forest and land management actors in Sumba are closely related to the Marapu local belief system. Marapu governs the way of life of indigenous Sumbanese society, including natural resource management. They ensure that there are sustainable ways to use natural resources. Farming practices and natural resource management are carried out in sustainable manners to please Marapu. Natural disasters or harvest failures are considered a result of the mismanagement of natural resources that make Marapu angry, and they should be solved by performing rituals to restore good relationships with Marapu ( Ngongo & Ngongo, 2021).

Local communities in East Nusa Tenggara have practiced the ecosystem services of community forests in the so-called Mamar in Timor and Kaliwu in Sumba. This typical local agroforestry has been established across the Wallacea region, with different local names; however, it has similar functions in integrating various compatible plants or vegetation in a parcel of land to create stable ecosystems, conserve biodiversity, and provide crops and timber for households.

The government (Envinment and Forestry Agency) has the power to influence society. In addition, the power of government actors is supported by bureaucratic politics ( Sahide et al., 2020b, 2020c). However, the power of local government units related to environmental protection seems biased toward “profit-taking” to enhance local government revenue, while local or traditional institutions are less empowered to take part in environmental protection ( Palar & Lengkong, 2022).

### Discourse of actors in community forest management

Various problems in reforestation are related to actors who have power where resources are concentrated to the detriment of forest-dependent communities ( Barr & Sayer, 2012). The Indonesian government has adopted an ecological restoration approach that focuses on landscape restoration, thus marginalizing socioeconomic and institutional factors, which are factors that influence the success of reforestation ( Indrajaya et al., 2022). On the other hand, reforestation policies are hampered by not considering the local context, which is related to information sharing, collective action, and conflict management mechanisms ( Prins et al., 2023; Sharma & Yonariza, 2021). Forest Management Units that are established may play pivotal roles as local intermediaries in resolving local conflicts, and as intermediaries between central and local governments for greater consistency in policy and implementations ( Fisher et al., 2017).

Traditionally, research on power has often concentrated on structural or institutional analysis, ignoring the vital role of individuals or groups in power dynamics ( Lawrence & Buchanan, 2017). An ACP approach in forest management is essential to understand how power is exercised, distributed, and used by the various actors involved. The ACP approach is an analytical tool to identify elements of power that are used to achieve specific political interests related to forest management ( Krott et al., 2014). Furthermore, this approach can uncover interests and powers that come in various forms and with missions that are hidden ( Prabowo et al., 2016) due to the influence and authority of actors who do not want to be known by competitors and researchers ( Maryudi & Fisher, 2020). Therefore, the ACP approach helps identify conflicts of interest, highlight factors that influence decisions, and formulate management strategies that involve the participation of different actors in a more inclusive manner. The ACP approach has also expanded with sequential power framework analysis that is useful for exploring equality and justice by highlighting advantaged and disadvantaged actors ( Sahide, Fisher, Supratman et al., 2020a; Sahide, Fisher, Verheijen et al., 2020c).

The ACP ideas were implemented during the devolution era in Indonesia, especially in agriculture, forestry, and natural resource-based management in general, including in the Wallacea region that covers most of eastern Indonesia. Considering the decline of forest areas, it seems that the ACP ideas are not reflected. Nevertheless, the facts have shown that these ideas are more conceptual but lacking in the commitment necessary to be applied, particularly by government apparatus and other actors that may benefit from the previous policy or during the New Order government. The ACP model is applied more for formal processes and to benefit the political actors ( Nurprabowo et al., 2021) and strengthen the existing powerful actors ( Sahide, Fisher, Supratman et al., 2020b), and the forestry governance system may endure and deepen in the post of Indonesia’s Job Creation Act (Omnibus Law) era ( Ramadhan et al., 2023). Since the devolution era, the local government has been encouraged to increase its local revenues while limiting the budget allocated for forest monitoring and protection. Power transitions during devolution are closely connected to Indonesia’s “chaotic” or social disorder era, particularly in land and natural resource-based management. In the case of the Wallacea Line in the western part of Timor Island, the forest quickly deteriorated due to the influx of refugees from East Timor, who mostly settled in or around forestland ( Ngongo, 2011).

Despite its high biodiversity, the Wallacea area is highly diverse regarding agroecosystems and people, making them interdependent in acquiring and sharing forestry products. For a long time, people in East Nusa Tenggara, especially in the towns, have depended highly on wood for building materials from Sulawesi Island. At the same time, East Nusa Tenggara provides livestock and palm sugar to people in Sulawesi and the surrounding islands. This traditional interdependence has been disturbed, since the central government now regulates or limits wood trading for forest protection. Limiting the inter-island wood trade has increased the price of wood for building materials, leading to a rise in the use of local wood and the cutting down of local trees.

Although the decentralization era started with the establishment of the Regional Autonomy Act 22/1999, the State actors have a monopoly on setting forest boundaries, as the forestland is under the authority of the Ministry of Forestry and Environment. Classified forestlands sometimes do not align with the local people’s beliefs. Local people of Timor, for example, claim that the Mutis forest or area is their traditional “fortress” and a central area for their livelihoods ( Ngongo, 2011; Ngongo & Markus, 2020); The Marapu belief system of the local people in Sumba Island considers the forestland and all natural resources within it as a sacred “unity ecosystem” and that before extracting any materials from it, permission should be asked from God through a ceremony ( Ngongo & Ngongo, 2021). The same principles are applied by the Indigenous people of Tana Toraja who contributed to the success of forest conservation ( Hardjanto & Patabang, 2019). The local knowledge and belief system of the Toraja people do not allow them to cut down trees or slaughter animals indiscriminately ( Naping, 2007).

## Conclusion: Proposed Model of Sustainable Forest Management in the Wallacea Region

The ACP approach, which aligns with the spirit of devolution and regional autonomy in Indonesia, should be operationalized to protect and conserve the Wallacea ecosystem. The actors involved should not be locked in the government’s administrative boundaries but should rather be boundless or involve broad integrative and social-cultural networks long established in the region. The biophysical diversity of the Wallacea region, the people making a living in it, and all actors involved, particularly from government levels (national, provincial, and district), should be in harmony and flexible in developing communications for strengthening and effectively implementing protections and conservation programs in the Wallacea Line.

The main aspects for improvement are solid and clear links between the central government and local governments (provincial and district) in collaboration with local/community actors for forest governance. Besides, there should be a solid willingness to apply law enforcement in forest protection. Cooperation and program synchronization among central government ministries and agencies are essential in planning, program implementation, and evaluation to ensure that implemented programs are mutually supportive in forest and natural resource protection while broadening people’s participation and improving the livelihoods of local communities.

**Figure 7.**
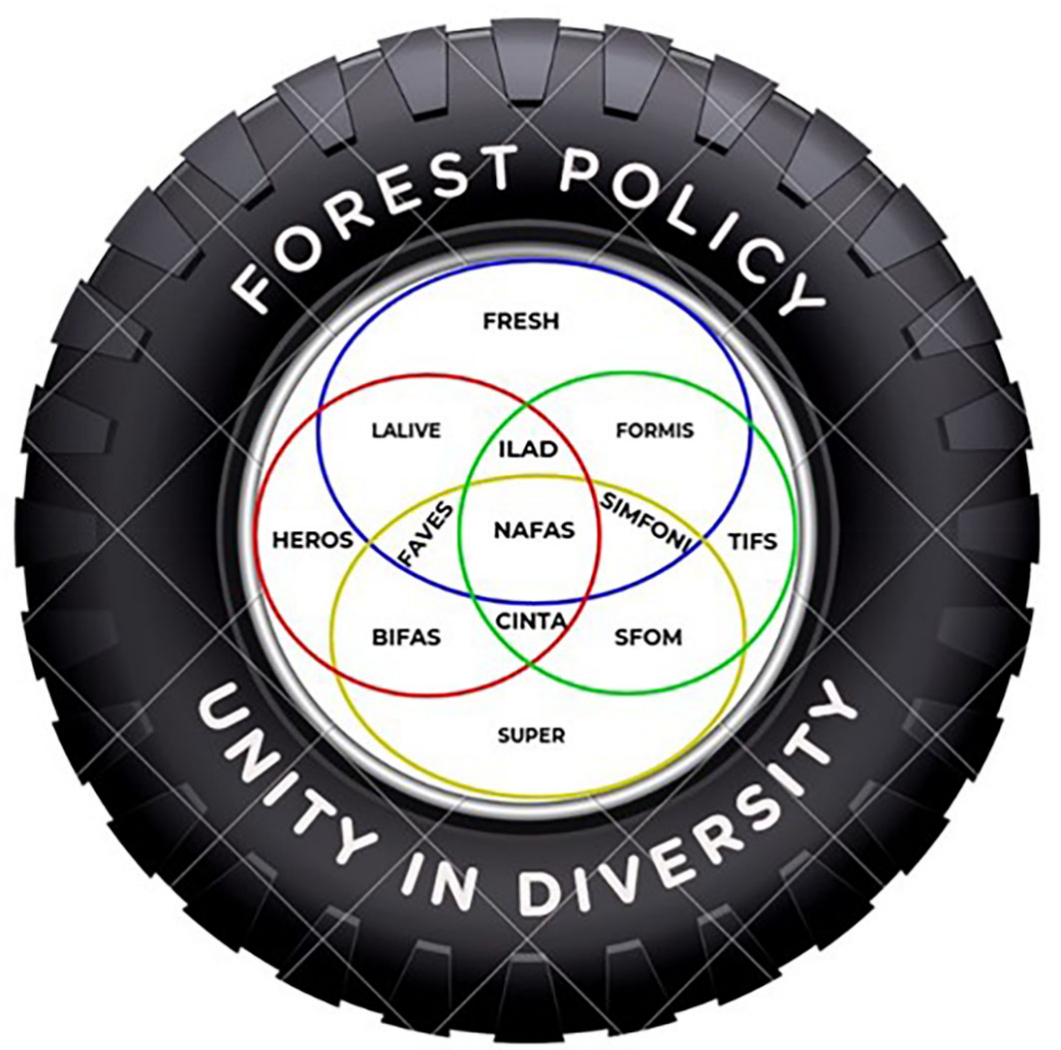
Proposed Model of Wallacea Sustainable Forest Policy.

Considering the uniqueness of the Wallacea region, we propose a model ( Figure 7) where all regional actors can work together in harmony with local institutions and communities to protect the Wallacea ecosystem. The Wallacea region should be seen as a unified ecosystem that is not limited to bureaucratic and government administrative boundaries. As the Wallacea region is located across several provincial administrative boundaries in eastern Indonesia, according to Government Regulation No. 32/2009 concerning environmental protection and management, it should be under the central government authority.

As the Wallacea region is a unique bioregion ecosystem, it requires an extraordinary institution across government institutions and administration boundaries to oversee biodiversity protection policy/programs. The proposed institution will harmonize national legislation and policy with the regional (provincial and district) institutions and make sure there are no overlaps with the local institutions and legislations in implementing the programs/policy. The proposed institution will also bridge the collaborations with the international communities or institutions concerned with the Wallacea region.

Within this unified ecosystem, the interdependence of local communities in managing and using local resources to make a living will be considered. Traditional inter-island trading for local or indigenous commodities among local communities that have long respected the local resources and sustainable practices will be revitalized. The proposed institution will oversee and integrate those customary practices in natural resource use and inter-island trade into modern or national trading systems.

The destruction of forests and natural resources in the Wallacea ecosystem results from weakening law enforcement commitments, segmentation, and simplifying the role of actors involved in sustainable forest management. We propose a forest management policy based on understanding social, cultural, and geographical diversity in forest management as a reflection of the basic principle of “unity in diversity.” Interests in forest resources are necessary, so every actor from the government, the private sector, and the community has various interests that must be aligned with sustainable forest resource management. In other words, every actor at the central, provincial, and district levels should reposition their power, capacity, and interests to drive togetherness to achieve sustainable forests for prosperous communities. Therefore, the wheel of forestry policy should be able to arrange interdependence between interrelated and dependent actors on the same forest resources. The characteristics of Wallacea’s diverse forest ecosystem require a powerful policy wheel in all situations and challenges, ensuring legal certainty and facilitating the development of local forest management initiatives specific to the Wallacea ecosystem. Specific forest management in the Wallacea ecosystem is a necessity and a tremendous opportunity. It is based on an integrated fundamental understanding of the Nature of Forest Attributes and Services (NAFAS), covering sociocultural, economic, ecological, and ecosystem service aspects. NAFAS is supported by four main pillars: (1) Biodiversity Indicators of Forest Attributes & Services (BIFAS), which prioritize the protection and maintenance of biodiversity in the form of flora, fauna, and ecosystem services in Wallacea; (2) Sustainable Forest Opportunity & Management (SFOM), a beacon of hope for the future, which mainstreams a sustainable future for the Wallacea forest ecosystem through law enforcement and fire and deforestation mitigation; (3) Forest Resource Management & Information System (FORMIS), which prioritizes the use of forest resource technology and information as a baseline for the protection and utilization of the potential of forest resources and ecosystem services; and (4) Landscape Attribute & Livelihood Initiatives Environmentally (LALIVE), an integrated approach to landscape-based forest ecosystem management as a single ecosystem, where actors take different roles to ensure sustainable forest management and utilization. With these pillars in place, we can look forward to a future where the Wallacea ecosystem thrives, and its resources are managed sustainably for the benefit of all.

Each pillar of NAFAS is connected through a wedge of interests that need to be understood and used as an indicator to understand the synergy and evaluation of forest resource management. Forest Asset & Value of Ecosystem Services (FAVES) is an indication of how the performance of actors is evaluated based on the synergy built on the BIFAS & LALIVE pillars, which ultimately boils down to Human Empowering, Resource Opportunity, and Skills (HEROS), which has direct implications for the sociocultural benefits of forest management. On the other hand, Criteria, Indicators & Target Achievements (CINTA) is a slice of BIFAS and SFOM that represents biodiversity management in the form of flora, fauna, and the ecosystem, as well as having implications to ensure the future of forest resource management, which leads to Sustainable of Prosperity (SUPER) for the socio-economic function of forests for interested actors. On the other hand, System Information Management of Forest & Nature Indicators (SIMFONI), as a wedge of synergy between SFOM and FORMIS, emphasizes that the management of natural resources and forest information is carried out in an integrated manner through several criteria and indicators that can be used together by all parties in forest resource management. In turn, it will lead to Technology Information of Forest Situation (TIFS) as a means of information that is easily accessible to all parties to understand the dynamics of forest management and utilization and biodiversity that have implications for socio-economic and environmental vulnerabilities. Likewise, Integrated Landscape Area Development (ILAD) is a synergy between FORMIS and LALIVE, an integrated approach that synergizes para-actors and their interests in measurable and sustainable forest management. The synergy of FORMIS, LALIVE, and ILAD encourages the creation of Forest Resource Ecological Sustainability & Harmony (FRESH), which refers to harmonizing interests and regulations among actors to avoid sector regulations that have been overlapping, thus supporting the goals of sustainable forest management and prosperous communities.

We contend that our proposed concept differs from the previous concept, which places actors as the principal elements and is separate from each other based on their interests and sectors. In this concept, the actors are positioned as a unit of the driving chain of forest ecosystem management dynamics. Actors need to be placed as a subsystem of the entire sustainable forest management system so that the success of forest management results from the intervention of actors and the carrying capacity of the forest ecosystem. Thus, the principle of unity in diversity in forest management policies must provide space for the growth of creativity and positive contributions from each actor entity with its power and authority as one of the links that drive the wheels of forest management policies to be carried out in an integrated and sustainable manner at every opportunity and challenge.

Thus, forest management policies may change according to needs over time, and actors will experience changes in orientation and interests. Still, the NAFAS central circle and its supporting pillars as the primary drivers of forest management policy, as well as a filter for each actor involved, must not undergo significant changes because, in fact, forest sustainability is the determinant of the sustainability of its benefits to actors and the balance of the ecosystem.

## Ethical consideration

Ethical approval and consent were not required.

## Data Availability

No data are associated with this article.
