# Supplementary material for: Engaging Sustainable Reforestation and Forest Protection in the Wallacea Line, Indonesia
Source: F1000Res. 2025 Sep 8;14:165. Originally published 2025 Feb 5. [Version 3] doi: 10.12688/f1000research.159731.3 (PMC12498518; doi:10.12688/f1000research.159731.3)
Supplement: Peer review history

# Reviewer report: Reviewer response for version 3

**DOI:** 10.5256/f1000research.187340.r412223

**Published:** 2025-09-26

**Authors:** Ambika P. Gautam

**Affiliations:** 1 Kathmandu Forestry College, Chandragiri, Nepal

I am satisfied with the revision.

Is the review written in accessible language?

Yes

Are all factual statements correct and adequately supported by citations?

Yes

Are the conclusions drawn appropriate in the context of the current research literature?

Partly

Is the topic of the review discussed comprehensively in the context of the current literature?

Partly

Reviewer Expertise:

NA

I confirm that I have read this submission and believe that I have an appropriate level of expertise to confirm that it is of an acceptable scientific standard.

# Reviewer report: Reviewer response for version 2

**DOI:** 10.5256/f1000research.182667.r398298

**Published:** 2025-07-26

**Authors:** Faisal Ismail Musa

**Affiliations:** 1 Forestry, University of Blue Nile, Ad-Damzin, Sudan; 2 Forestry, Mizoram University (Ringgold ID: 29670), Aizawl, India

Manuscript under title (Engaging Sustainable Reforestation and Forest Protection in the Wallacea Line, Indonesia), by Ngongo et al.,

 This is a well-structured and richly contextualized manuscript addressing critical issues surrounding sustainable reforestation and forest protection in the Wallacea bioregion. The authors present a comprehensive analysis using the Actor-Centered Power (ACP) framework and propose a governance model grounded in regional cooperation and sociocultural embeddedness.

 However, this are some points need to be address by author to more clear to the readers.

 1. There is no clear methodology followed by author to in conducting the study such as sample size, how these actor identifies ....etc.

 2. Clarity and Focus on ACP Application: While the manuscript asserts the relevance of the ACP approach, its empirical application remains largely descriptive and generalized. I recommend a clearer operationalization of ACP dimensions (coercion, incentives, information) across specific actor interactions and case sites (e.g., Matalawa, Mutis). Inclusion of structured comparison or actor-mapping tables would improve analytical depth.

 3. Evidence of Policy Impact: The manuscript highlights numerous policies and initiatives, including social forestry, TORA, and decentralization, but lacks robust evidence on their differential outcomes. Consider incorporating more specific indicators (e.g., forest cover change, community income shifts, conflict mitigation) to evaluate the impact of these programs.

 4. Model Validation: The proposed forest governance model (Figure 7) is a valuable conceptual contribution, but further elaboration is needed on how it can be tested, implemented, or scaled within existing legal and institutional frameworks.

 5. the new proposed model in conclusion can be separated from the conclusion.

 6. check the references in and out text and should be matching.

Is the review written in accessible language?

Yes

Are all factual statements correct and adequately supported by citations?

Yes

Are the conclusions drawn appropriate in the context of the current research literature?

Yes

Is the topic of the review discussed comprehensively in the context of the current literature?

Yes

Reviewer Expertise:

Agroforestry, forest management, forest economic, regeneration ecology ... etc

I confirm that I have read this submission and believe that I have an appropriate level of expertise to confirm that it is of an acceptable scientific standard, however I have significant reservations, as outlined above.

# Reviewer report: Reviewer response for version 2

**DOI:** 10.5256/f1000research.182667.r396117

**Published:** 2025-07-22

**Authors:** Ambika P. Gautam

**Affiliations:** 1 Kathmandu Forestry College, Chandragiri, Nepal

The manuscript reflects extensive research and understanding of both historical and contemporary issues related to deforestation, governance, and community involvement in forest management in the Wallacea region of Indonesia. The research provides valuable insights into the ecological and socio-economic importance of the region while also discussing the challenges and opportunities for sustainable forest management. It contributes to enhancing understanding of how effective policy and suitable management approach is necessary to achieve conservation objectives and overall environmental sustainability.

 Introducing the actor-centered power (ACP) approach provides an interesting framework for analyzing the complexities of forest management. It demonstrates the authors' intention to merge theoretical concepts with practical implications, which enriches the study. Overall, this document is an important contribution to the discourse on sustainable forestry in Indonesia, particularly in the Wallacea region.

 The manuscript, however, has some weaknesses and gaps (see below), which need to be addressed before it is considered for publication.

**Introduction:**

 The introduction, which is focused mainly on describing community-based forest management in Indonesia, should be revised by providing clear background on Wallacea's unique biodiversity, and problems with the current forest management systems. The section should more effectively set the stage for the urgency of the need for reforestation and state the research problem more clearly.

**Study area:**

 A brief introduction to the study area, along with a location map, would be helpful to better understand the ecological and socio-economic context of the Wallacea region. The manuscript does include some information about the region here and there (e.g. p 9) but it is not well organized. It would be better to briefly describe the study area before presenting the results.

**Methodology:**

 Lack of information on the research methodology is a major gap. The manuscript does not provide clear information on the types of data used, and methods of data collection and analysis. Is it purely a review-based paper? If yes, what approach was taken in literature collection, review, and analysis? Brief information clarifying these aspects would be helpful for the readers.

**Cocoa discourse:**

 The subsection of cocoa plantations highlights the duality of economic development versus environmental degradation. A more detailed exploration of agroforestry as a solution would further strengthen this section.

**Conclusion:**

- The conclusion summarizes key points well but it is not well supported by the data/findings. How can the authors confidently conclude that the ACP approach, which is claimed to be successful in some other areas with different ecological and socioeconomic contexts, would be successful in the Wallacea region that has a unique context?

- The authors may like to include a section on potential future research directions or areas that require further investigation to address existing gaps in knowledge regarding forest management in the Wallacea region.

**Other comments:**

- The principle of “unity in diversity” is intriguing but lacks sufficient explanation. Providing a brief description or example of how this principle could be applied in the proposed sustainable model would be useful.

- The inclusion of traditional ecological knowledge, particularly how indigenous practices contribute to biodiversity conservation, might offer additional perspectives on sustainable forest management.
- How is the “critical land” defined in the research (Fig. 2)?
- What is meant by “land fires” in this research (Fig. 3)? Do these include both forest and agricultural land fires or only forest or wildfires?

Is the review written in accessible language?

Yes

Are all factual statements correct and adequately supported by citations?

Yes

Are the conclusions drawn appropriate in the context of the current research literature?

Partly

Is the topic of the review discussed comprehensively in the context of the current literature?

Partly

Reviewer Expertise:

Forestry, Forest ecology, biodiversity and ecosystems, community based forest management, NRM strategies, human dimensions of changes in biophysical environment

I confirm that I have read this submission and believe that I have an appropriate level of expertise to confirm that it is of an acceptable scientific standard, however I have significant reservations, as outlined above.

# Reviewer report: Reviewer response for version 1

**DOI:** 10.5256/f1000research.175499.r366603

**Published:** 2025-03-03

**Authors:** Johan Kieft, simon f

**Affiliations:** 1 climate chnage division, UNEP, NAIROBI, gigiri, Kenya; 2 Land Use and Green Economy, Indonesia, Indonesia; 3 management, besi pea foundation, soe, NTT, Indonesia

The article has not looked into reports on social forestry, including communal forests on deforestation as they show that social forests had no aggregate deforestation reductions from the rollout of community land titles in Indonesia, yet.  they should identify how their approach addresses this.  in addition, fire is an important aspect in particular on Timor and the Sunda. For Sulawesi, cacao has been a key driver of deforestation, even more than palm oil.  in addition, the impact of nitrogen-fixing trees has had an effect in Timor and Sumba on stabilizing tree cover.

Is the review written in accessible language?

Yes

Are all factual statements correct and adequately supported by citations?

Partly

Are the conclusions drawn appropriate in the context of the current research literature?

Partly

Is the topic of the review discussed comprehensively in the context of the current literature?

Partly

Reviewer Expertise:

deforestation,  climate finance, land, degradation, climate risk management, integrated fire mansgement

We confirm that we have read this submission and believe that we have an appropriate level of expertise to confirm that it is of an acceptable scientific standard, however we have significant reservations, as outlined above.

## References

[1] : Forage tree legumes and sustainable upland farming in Timor Island – Indonesia. *IOP Conference Series: Earth and Environmental Science* .2021;807(3) : 10.1088/1755-1315/807/3/032035 10.1088/1755-1315/807/3/032035

[2] : Deforestation on the Indonesian island of Sulawesi and the loss of primate habitat. *Global Ecology and Conservation* .2020;24: 10.1016/j.gecco.2020.e01205 10.1016/j.gecco.2020.e01205
